# Validation of Rapid Enzymatic Quantification of Acetic Acid in Vinegar on Automated Spectrophotometric System

**DOI:** 10.3390/foods9060761

**Published:** 2020-06-09

**Authors:** Irene Dini, Ritamaria Di Lorenzo, Antonello Senatore, Daniele Coppola, Sonia Laneri

**Affiliations:** 1 Department of Pharmacy, University of Naples Federico II, Via Domenico Montesano 49, 80131 Napoli, Italy; ritamaria.dilorenzo@unina.it (R.D.L.); srlaneri@unina.it (S.L.); 2 Lcm Laboratory for Product Analysis of Chamber of Commerce, Corso Meridionale, 80143 Napoli, Italy; a.senatore@si-impresa.na.camcom.it (A.S.); d.coppola@si-impresa.na.camcom.it (D.C.)

**Keywords:** vinegar, automatized method, quantification

## Abstract

Vinegar is produced from the fermentation of agricultural materials and diluted acetic acid (diluted with water to 4–30% by volume) via sequential ethanol and acetic acid fermentation. The concentration of acetic acid must be measured during vinegar production. A Community method for analyzing acetic acid in vinegar is a non-specific method based on the assumption that the total acid concentration of the vinegar is attributable to the acetic acid. It consists of titration with a strong base in the presence of an indicator. This test is laborious and has a time-consuming character. In this work, a highly specific automated enzymatic method was validated, for the first time, to quantify the acetic acid in the wine vinegar, in terms of linearity, precision, repeatability, and uncertainty measurement. The results were compared to the Community method of analysis. Regression coefficient ≅ 1 and the normal distribution of residuals in the ANOVA test confirmed the method’s linearity. LLOD (0.946 ppm) and LLOQ (2.00 ppm) defined the method’s sensitivity. The results of the tested and the Community methods, linearly distributed in the Shapiro–Wilk test, confirmed the method’s repeatability. The few anomalous data in the Huber test were due to random errors. The high selectivity of the enzymatic method, which exclusively measures acetic acid concentration, determined the significant differences between the two tests, examined in the accuracy determination. The enzymatic method can be considered applicable since its precision and uncertainty were lower than the Community method values (relative percentage deviations = 10%). The enzymatic method compared to the Community method reduces the analysis time and the risk of errors due to operators (avoid pipetting errors and wrong calculations), minimizes solvent and the sample consumption and guarantees assay quality through method standardization.

## 1. Introduction

In the European Member States, products obtained by the fermentation of agricultural materials or by the dilution with water of acetic acid are marketed under the name “vinegar” [1]. According to the raw material used in production, there are many types of vinegar: wine, cider, fruit, malt, malt distillate, spirit, cereal, honey, and whey vinegar. Wine vinegar is widely used as a seasoning, food preservative, and acidifier. Traditional production needs maturation for a long time in the wood to obtain a high acetic degree. Two stages of fermentation lead to the production of wine vinegar. In the first step, the yeasts, generally Saccharomyces, convert the fermentable sugars into ethanol. In the second phase, the bacteria oxidize the ethanol to acetic acid [2]. In Italy, three types of wine vinegar are produced: white wine vinegar, red wine vinegar, and balsamic vinegar. The latter is obtained from fresh grapes, concentrated by a slow heating process (to 1/3 of its original volume), fermented by yeasts (*Zygosaccaharomyces*) and bacteria (*Gluconobacter*), and subsequently refined in wooden barrels (25 years) [3]. Acetic acid is monitored during the acetic fermentation process. A Community method for the analysis of acetic acid consists of direct titration with sodium hydroxide in the presence of phenolphthalein [4]. This method is not selective; it determines the total acidity of the vinegar and attributes it to the content of acetic acid. Titration is an analytical methodology that uses color to measure the quantity of substances. The visual identification of the endpoint can lead to quantification errors. Therefore, accuracy, sampling frequency, and time expenditure are difficulties generally associated with manual titrations. The alternative methods proposed to determine acetic acid in vinegar are spectrophotometry with a fiber optic sensor [5], a titration system with colorimetry (λ480 nm) [6] or an ATR-FT-IR detector, a chemometric test [7], capillary electrophoresis or ion exclusion chromatography with conductimetric detection, [8,9] and liquid chromatography and gas chromatography [10,11]. In this work, we propose the validation of an automated enzymatic method to identify and quantify acetic acid in vinegar. The automated analyzer was designed to disperse the reagents and samples in the cuvette, incubate the samples at a controlled temperature, read the absorbance in the UV-visible spectrum, and calculate the concentrations of the selected molecules using a calibration curve. The highly selective enzymatic reaction allows the detection of acetic acid in spectrum fields without interference. Following regulatory requirements, the validation of the method is essential to establish data traceability and avoid incorrect quantification, which could have economic consequences and damage the reputation of the laboratories. The validation exercise is expensive and time-consuming. It would be desirable for the scientific community to spend more time validating advanced analytical methods for food quality control [12] to eliminate test repetitions and avoid wasting time.

## 2. Materials and Methods

### 2.1. Reagents

Enzytec acetic acid Cod. E2580 was purchased from R-Biopharm AG (Darmstadt, Germany). Distilled water was purchased from Sigma-Aldrich (Milan, Italy). Potassium hydrogen phthalate and ethanol were purchased from Carlo-Erba (Milan, Italy).

### 2.2. Samples Preparation

Three commercial vinegar types were tested: white, red, and balsamic wine vinegar. Samples were diluted 1:125 before analyses.

### 2.3. Apparatus

The analyzer iCubio iMagic M9 was used and run with full automation for the enzymatic assay for acetic acid determination. It automatically pipetted reagents and samples into the cuvette, allowed incubation at a controlled temperature, read absorbance at the specific wavelength, and calculated the concentration of the analytes with a calibration curve. The parameters used in the automated photometric systems were temperature, 37 °C; wavelengths, 340 nm and 415 nm (bichromatic); and optical path, 1 cm.

### 2.4. Reference Procedure

Commercial vinegar samples were analyzed by titration to determine the acetic acid content following the Community reference method [12]. A NaOH solution, normalized with potassium hydrogen phthalate (ACS), was gradually added to 5 mL of the vinegar solution. Complete neutralization was indicated by color changes in 2% phenolphthalein solutions in ethanol. Triplicate analyses were carried out. The average of the three analyses was used as the reference value. The mean standard error (pooled standard deviation divided by the average acidity content) was 0.32%.

### 2.5. Enzymatic Method Determination of Acetic Acid Content

The method reported in the kit instruction (Enzytec acetic acid) was followed. The Enzytec fluid Acid combination Standard (ID-No 5460, 3 × 3 mL) was used to calibrate the automated photometric systems.

### 2.6. Spectrophotometric Method Validation Parameters

The linearity, precision, sensitivity, and measurement uncertainty were determined.

In the linearity assessment, a matrix-match calibration curve was plotted at 0, 0.06, 0.13, 0.25, and 0.50 g/L for acetic acid.

The method precision was tested, performing ten analyses of the same sample

(1) $$\mathrm{Reproducibility}=\frac{\mathrm{Standard}\;\mathrm{deviation}\;\mathrm{of}\;\mathrm{analyzed}\;\mathrm{samples}}{\mathrm{Standard}\;\mathrm{deviation}\;\mathrm{of}\;\mathrm{reference}\;\mathrm{samples}}$$

Normality, by the Shapiro–Wilk test [13], and the presence of anomalous data, by the Huber test [14], were evaluated to define the method’s precision.

The method sensitivity was evaluated by LLOQ (limit of quantification: LOQ = 10σS) and LLOD (limit of detection: LOD = 3.3σS) determinations, where σ is the relative standard deviation and S is the slope of the standard curve.

The method accuracy was determined, performing ten analyses with both methods (Community [4] and enzymatic), determining the residual distribution by the Saphiro–Wilk test, and controlling for anomalous data by *t*-tests.

Type A and B uncertainties were measured following the EURACHEM/CITAC guide [15]. Type A was estimated from 10 repeated readings of the same sample.

(2) $$U\;\mathrm{Type}\;A=\sqrt{\frac{\mathrm{variance}}{\mathrm{Degrees}\;\mathrm{of}\;\mathrm{freedom}}}$$

Type B was determined with a metrology approach.

U(t) is the uncertainty associated with 20 mL pipette use U(t). It was obtained considering a certificate of calibration (0.016 mL) and repeatability (0.00050 mL).

U(p) is the uncertainty associated with 10 mL pipette use. It was obtained considering a certificate of calibration (0.096 mL) and repeatability (0.00020 mL).

U(ct) is the uncertainty associated with the calibration curve. It was obtained for the standard, which was measured at three concentrations in triplicate.

(3) $$U(\mathrm{ct})\;S\frac{x/y}{b}\;*\sqrt{1/n+1/m\;}$$

S = standard deviation of the residual

n = points used for the calibration line

m = readings taken for each sample

U(mr) is the uncertainty associated with a standard preparation.

U(bt) is the uncertainty associated with balances. It was determined considering a certificate of calibration (0.00060 g), repeatability (0.000029 g), and stability (0.000032 g).

U(m) is the uncertainty associated with the use of a 100 mL flask. It was obtained considering a certificate of calibration (0.01 mL) and repeatability (0.00030 mL).

U(k) is the uncertainty associated with the use of a 250 mL flask. It was obtained considering a certificate of calibration (0.025 mL) and repeatability (0.00040 mL).

Compound uncertainty: $\sqrt{{\left(U\;Type\;A\right)}^{2}+{\left(U\;Type\;B\right)}^{2}}$

The accuracy was tested by Student’s *t* test:

(4) $$\mathrm{Accuracy}=\;\frac{\left|{\bar{X}}_{\mathrm{Community}}-\bar{\;X}\right|}{\sqrt{S_{r}^{2}+U_{\mathrm{Community}}^{2}}}\leq {{t_{p}}_{\nu }}_{\;}$$

$\bar{X}$ = Community method value

$\bar{X}$s_y/x_ = medium repeatability values

$S_{r}^{2}$ = standard deviation^2^

$U_{\mathrm{CRM}}^{2}$ = reference material uncertainty^2^

Uncertainty (*t*_pν_) = k × σr.

### 2.7. Statistical Analysis

The data were analyzed using the software Statistica Version 7.0 (StatSoft, Hamburg, Germany). The normality of the data was verified by applying the Shapiro–Wilk *W*-test. A *p*-value of >0.05 indicates a normal distribution. The non-parametric Huber test determined the outliers.

## 3. Results

### 3.1. Method Linearity

The method linearity, by regression coefficient determination (Figure S1), and residuals distribution, by ANOVA tests (Figure S2), were evaluated. R^2^ = 0.99; the residuals were normally distributed.

### 3.2. Method Sensitivity

The method detection limit was tested by repeated analyses of blank samples. The LLOD and LLOQ were derived from the regression curve. The quantitation limit (LLOD) of an individual analytical procedure is the lowest amount of acetic acid in a sample that can be quantified with suitable precision and accuracy. The lower limit of detection was 0.0063 g/L. The detection limits (LLOQ) were determined as the concentration giving a peak height three times the background noise. The Lower Limit of Quantitation was 0.0253 g/mL. The LLOQ dilution factor was used to determine the lower end of the measuring range. It was obtained by dividing the (read concentration × 10) / (weight × rate) (0.098 g/100 mL). The last point of the calibration curve line was the upper end of the measuring range (0.40 g/100 mL).

### 3.3. Measurement Uncertainty

Type A uncertainty due to method repeatability was 0.005 (Table S1). Type B uncertainties—due to method repeatability and associated with the standard preparation, the calibration curve, the balances, the flasks (100 mL and 250 mL), and the pipettes (10 mL and 20 mL)—were less than 10% of the results (Table S2).

### 3.4. Method Precision

Method repeatability was tested, making ten analyses on the same sample of each type of vinegar and comparing the results with those obtained with the Community method. The Shapiro-Wilk test showed that the data were linearly distributed (Figure S4). The Huber test excluded the presence of anomalous data (Figures S3–S5). The reproducibility of the three kinds of vinegar were red wine vinegar = 0.55, white wine vinegar = 0.69, and balsamic wine vinegar = 0.66. The limits of method repeatability were: upper limit = 0.548 and lower limit = 1.480, considering the ratio between the standard deviation (Sr) of the enzymatic method and the repeatability standard deviation of the reference method (σr), satisfied for nine degrees of freedom (Table S6).

### 3.5. Accuracy Test

Accuracy was determined, making ten analyses with both methods (Community and enzymatic), and determining significant differences between groups by Student’s *t* test (Figures S4–S6). We cannot reject the null hypothesis that there is no difference between means when *p* < 0.05.

## 4. Discussion

Automated analyzers are modern instrumentation for routine analytical analysis since they reduce staff errors due to tiredness or a lack of technicality, improve safety, and decrease the amount of reagents, the cost, and the time of analysis. Traditional methods of analysis are struggling to survive in technology. Innovation in technologies brings significant opportunities but also carries risks for society. The validation of new analytical procedures is a developed approach that responds to evolving markets. It is a verification process that checks whether the analytical method achieves predetermined results. The validation of an analytical procedure is used both before its first use and throughout its life, to continually monitor its performance and any critical issues. In food, analysis is indispensable to make available reliable and accurate results with known uncertainty. Therefore, the methods used in analytical laboratories need an accurate validation process to ensure their validity (ISO/IEC, 2005) [16]. Validation is performed to define the linearity, precision, accuracy, and repeatability of the method based on the matrix, the working field, and the uncertainty due to the instrumentation and environmental conditions. Furthermore, it is possible to verify the results by comparing them with those obtained with reference analytical methods. In this work, an enzymatic determination of acetic acid in three different types of vinegar (red wine vinegar, white wine vinegar, and balsamic vinegar) was carried out on automated photometric systems. The method was based on acetate kinase, an enzyme capable of reacting with acetic acid and adenosine-5’-triphosphate, giving acetylphosphate and adenosine-5’-diphosphate (ADP). Acetylphosphate is converted into acetyl-CoA plus phosphate by coenzyme A (CoA) and phosphotransacetylase. ADP reacts with D-glucose through an ADP-dependent exokinase to produce D-glucose-6-phosphate. The latter, in the presence of glucose-6-phosphate dehydrogenase, reacts with NAD^+^, turning into D-glucono-δ-lactone-6-phosphate and NADH^+^ H ^+^. The concentration of NADH, proportional to the concentration of acetic acid, is determined spectrophotometrically, according to AOAC instructions (AOAC 2012) [17]. The test uses a kit containing ready-to-use reagents and standards. The analytical problem consisted of adapting the procedures proposed by the industry for the wine matrix to the vinegar matrix and the validation of the analytical procedures. The concentration of the solution influences the spectrophotometric reading. The vinegar samples were diluted 125 times to be able to read the absorbance at the desired wavelength. Any change requires an evaluation of the method’s performance. The validation of the method, comparative tests with standard methods, and co-validation between laboratories are the possible strategies to achieve this goal [18]. In this case, the method was validated in terms of the linearity, precision, repeatability, measurement of uncertainty, and accuracy. The linearity of the method was demonstrated by the regression coefficient (1) and a residual diagram (straight line) in the ANOVA test. The ANOVA test describes the difference in the standard deviations of the values obtained from the reference compared to the expected values. The reliability of the test depends on the normal distribution of residues with a 95% confidence level. The sensitivity of the method was defined by deriving the LLOD (0.946 ppm) and LLOQ (2.00 ppm) from the regression curve and determining the measuring range (100 ppm ≤ measuring range ≤ 500 ppm). The precision of the method was determined, confirming its reproducibility and repeatability. Repeatability produces the minimum precision value. It was obtained, making ten analyses of the same sample in short intervals of time, by the same operator, in the same laboratory, with the same method and equipment and comparing the repeatability type difference of the method (sr) with that of the reference method (sr). The Shapiro–Wilk test confirmed the linearity between the results obtained by the spectrophotometric method and those obtained by the reference method. The null hypothesis (H0) states that two elements or series of elements are normally distributed. This hypothesis is satisfied if the p-value is higher than 0.05, as happens for the types of analysis carried out (Figure S4). Successively, the absence of anomaly data was tested by the Huber test (Tables S3–S5). The Huber test is a robust statistical method to identify outliers, which may invalidate the resulting analysis, by first fitting most of the data and flagging data points. The presence of very few anomalous data was attributed to random errors. Finally, random and systematic uncertainties were detected to establish method accuracy. Random errors are caused by unpredictable changes in the experiment due to environmental conditions and measuring instruments. The errors due to the instrument or its data-handling system, or the instrument being wrongly used by the experimenter cause systematic errors. In this work, the uncertainties due to method repeatability and associated with the standard preparation, the calibration curve, the balances, the flasks (100 mL and 250 mL), and the pipettes (10 mL and 20 mL) were determined to be irrelevant since they were less than 10% of the results. Finally, the accuracy of the enzymatic method was evaluated. The accuracy showed significant differences between the two populations of data. The enzymatic method underestimated the results because of systematic errors. The percentage difference was calculated, and it was observed that the systematic errors were independent of the matrix but were influenced by the measuring range (five samples had waste results around 10). The differences in measurements between the two tests were due to the high selectivity of the enzymatic method, which exclusively measured the concentration of acetic acid and non-specificity of the Community method, which attributed to the concentration of acetic acid all of the acids present in the sample. Consequently, the underestimation of the enzymatic method was expected. Therefore, the method could be considered applicable since the relative percentage deviations compared to the values obtained with the official method are around 10%.

## 5. Conclusions

An enzymatic method on an automated spectrophotometric system was used for the quantification of acetic acid in vinegar. The enzymatic method met many validation requirements (linearity, sensitivity, and uncertainties) but not accuracy. However, the method can be considered applicable as the precision and uncertainty are lower than those of the Community method (deviations are around 10%). The validation of instrumental analytical methods not yet used as conventional methods should find more space in the scientific literature. By transferring knowledge on operating methods, the repetition of long and laborious validation processes that involve high costs and waste of time will be avoided.

## Supplementary Materials

The following are available online at https://www.mdpi.com/2304-8158/9/6/761/s1, Figure S1: Standard calibration curve, Figure S2: Normal residual probability, Figure S3: Shapiro-Wilk test (5% significance levels), Figure S4: Balsamic wine vinegar accuracy statistical analyses, Figure S5: Red wine vinegar accuracy statistical analyses, Figure S6: White wine vinegar accuracy statistical analyses, Table S1: Data considering calculating type A uncertainty, Table S2: Type B: Systematic uncertainty estimates, Table S3: Red wine vinegar data, Table S4: Withe wine vinegar data, Table S5: Balsamic wine vinegar data, Table S6: Upper and lower limit of repeatability.
