# Supplementary material for: Validation of Rapid Enzymatic Quantification of Acetic Acid in Vinegar on Automated Spectrophotometric System
Source: Foods. 2020 Jun 9;9(6):761. doi: 10.3390/foods9060761 (PMC7353505; doi:10.3390/foods9060761)
Supplement: Supplementary file 1 [file foods-09-00761-s001.pdf]

Table 1. data considering calculating type A uncertainty.

X	Balsamic vinegar	Red wine vinegar	White wine vinegar
1	5.8	4.53	5.43
2	5.86	4.54	5.43
3	5.86	4.54	5.45
4	5.89	4.54	5.46
5	5.8	4.53	5.45
6	5.9	4.56	5.51
7	5.83	4.5	5.5
8	5.85	4.49	5.58
9	5.83	4.53	5.49
10	5.85	4.49	5.49
X_m	5.8470	4.5250	5.4820
Y=16100x+452			
Standard deviation	0.033349996	0.0237	0.0429
Relative deviation (s_r)	0.0057	0.0052	0.0078
S(y/x)	108	108	108
U(ct)	0.005	0.005	0.005

Table 2. Type B systematic uncertainty estimates.

Balsamic vinegar data considering calculating type B uncertainty							
	U(t)	U(p)	U(ct)	U(mr)	U(bt)	U(m)	U(k)
Type B uncertainty Xm	0.030	0.020	0.005123	0.100000	0.025660	0.10000	0.3000
Type B uncertainty Xm/radq	0.001479	0.005774	0.001479	0.028868	0.007407	0.028868	0.086603
The uncertainty u $(Xm)_{B/Xm}$	0.000074	0.001155	0.0000253	0.004937	0.000370	0.000289	0.000346
Resulting relative uncertainty u(y)/y	0.00765						
Resulting uncertainty u(y)	0.045						
Coverage factor k (2<k<3)	2						
Extended uncertainty U(y)	0.089						
**Red wine vinegar data considering calculating type A uncertainty**							
Type B uncertainty Xm	U(t)	U(p)	U(ct)	U(mr)	U(bt)	U(m)	U(k)
Type B uncertainty Xm/radq	0.030	0.020000	0.005123	0.100000	0.025660	0.100000	0.3000
Uncertainty u $(Xm)_{B/Xm}$	0.001479	0.005774	0.001479	0.028868	0.007407	0.028868	0.086603
	0.000074	0.001155	0.000327	0.006380	0.000370	0.000289	0.000346
Resulting relative uncertainty u(y)/y	0.00835						
Resulting uncertainty u(y)	0.038						
Coverage factor k (2<k<3)	2						
Extended uncertainty U(y)	0.076						
**White wine vinegar data considering calculating type B uncertainty**							
Type B uncertainty Xm	U(t)	U(p)	U(ct)	U(mr)	U(bt)	U(m)	U(k)
Type B uncertainty Xm/radq	0.030	0.020000	0.005123	0.100000	0.025660	0.100000	0.3000
Uncertainty u $(Xm)_{B/Xm}$	0.001479	0.005774	0.001479	0.028868	0.007407	0.028868	0.086603
	0.000074	0.001155	0.000270	0.005266	0.000370	0.000289	0.000346
Resulting relative uncertainty u(y)/y	0.00952						
Resulting uncertainty u(y)	0.052						
Coverage factor k (2<k<3)	2						

Extended uncertainty U(y)	0.104

Table 3. Red wine vinegar data.

	X		
1	4.53	Data number (n)	10
2	4.54	Media (Xm)	4.525
3	4.54	Variance (s_r^2)	0.000561111
4	4.54	Standard deviation (s_r)	0.023687784
5	4.53	*t* Student (ν=n-1; p=0.95)	2.262157163
6	4.56	Coefficient of variation ratio (CV_r%)	0.523486939
7	4.5	Minimum (min)	4.49
8	4.49	Maximum (max)	4.56
9	4.53	Range	0.07
10	4.49	Median	4.53
		Media-upper confidence limit (p=0.95)	4.54194522
		Media-lower confidence limit (p=0.95)	4.50805478
		Media-confidence interval (p=0.95)	0.01694522
		Degrees of freedom (ν=n-1)	9
		**Normal Distribution (Shapiro-Wilk test 5%)**	**Yes**
		**Outlier (Huber test 5%)**	**No**

Table 4. Withe wine vinegar data.

	X		
1	5.43	Data number (n)	10
2	5.43	Media (Xm)	5.479
3	5.45	Variance (s_r^2)	0.00207667
4	5.46	Standard deviation (s_r)	0.045570458
5	5.45	*t* Student (ν=n-1; p=0.95)	2.262157163
6	5.51	Coefficient of variation ratio (CV_r%)	0.831729481
7	5.5	Minimum (min)	5.43
8	5.58	Maximum (max)	5.58
9	5.49	Range	0.15
		Median	5.475
		Media-upper confidence limit (p=0.95)	5.511599142
		Media-lower confidence limit (p=0.95)	5.446400858
10	5.49	Media-confidence interval (p=0.95)	0.032599142
		Degrees of freedom (ν=n-1)	9
		**Normal Distribution (Shapiro-Wilk test 5%)**	**Yes**
		**Outlier (Huber test 5%)**	**No**

Table 5. Balsamic wine vinegar data.

	X
1	5.8
2	5.86
3	5.86
4	5.89
5	5.8
6	5.9
7	5.83
8	5.85
9	5.83
10	5.85

Data number (n)	10
Media (Xm)	5.847
Variance (s_r^2)	0.001112222
Standard deviation (s_r)	0.033349996
t Student (ν=n-1; p=0.95)	2.262157163
Coefficient of variation ratio (CV_r%)	0.5703779
Minimum (min)	5.8
Maximum (max)	5.9
Range	0.1
Median	5.85
Media-upper confidence limit (p=0.95)	5.87085715
Media-lower confidence limit (p=0.95)	5.82314285
Media-confidence interval (p=0.95)	0.02385715
Degrees of freedom (ν=n-1)	9
**Normal Distribution (Shapiro-Wilk test 5%)**	**Yes**
**Outlier (Huber test 5%)**	**No**

Table 6. The upper and lower limit of repeatability.

Degrees of freedom (ν=n-1)	A		B
1	0.0316		2.241
2	0.160		1.921
6	0.454	$A \leq \frac{\text{Standard deviation of analyzed samples}}{\text{Standard deviation of reference samples}} \leq \mathbf{B}$	1.551
9	0.548		1.480
11	0.589		1.412
20	0.692		1.307
30	0.748		1.251

Figure 1. Standard calibration curve.

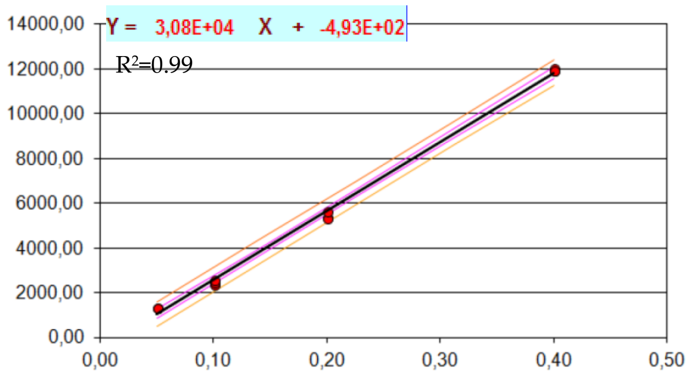

X	Y	Ycalc
0,05	1295,3214	1,05E+03
0,1	2342,5698	2,59E+03
0,2	5355,2365	5,67E+03
0,4	11900,0324	1,18E+04
0,05	1292,4565	1,05E+03
0,1	2468,0978	2,59E+03
0,2	5340,1236	5,67E+03
0,4	12028,3265	1,18E+04
0,05	1288,0232	1,05E+03
0,1	2536,9523	2,59E+03
0,2	5652,2132	5,67E+03
0,4	11901,0521	1,18E+04

Figure 2. Normal residual probability.

ID	X	h_i	e_i	e_{Ni}	e_{Si}	e_{Ji}	Studentized residuals <=1s	> 1s e <= 2s	> 2s
							<= 1,069	> 1,069 <= 2,139	> 2,139
1	5,00E-02	0,171	2,48E+02	1,094	1,202	1,233		x	
2	1,00E-01	0,119	-2,45E+02	-1,085	-1,155	-1,177		x	
3	2,00E-01	0,084	-3,13E+02	-1,384	-1,446	-1,543		x	
4	4,00E-01	0,293	7,07E+01	0,312	0,371	0,355	x		
5	5,00E-02	0,171	2,45E+02	1,082	1,188	1,216		x	
6	1,00E-01	0,119	-1,20E+02	-0,530	-0,564	-0,544	x		
7	2,00E-01	0,084	-3,28E+02	-1,451	-1,516	-1,639		x	
8	4,00E-01	0,293	1,99E+02	0,879	1,046	1,051	x		
9	5,00E-02	0,171	2,40E+02	1,062	1,166	1,190		x	
10	1,00E-01	0,119	-5,10E+01	-0,225	-0,240	-0,228	x		
11	2,00E-01	0,084	-1,62E+01	-0,072	-0,07[illegible]	-0,071	x		
12	4,00E-01	0,293	7,17E+01	0,317	0,3[illegible]	0,360	x		
13									
s(y/x)			2,16E+02	0,953463	1,030686	1,06933	6	6	0

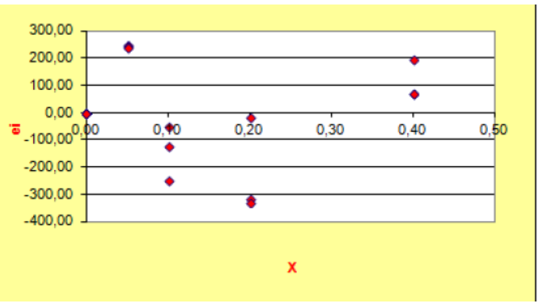

Absolute residuals

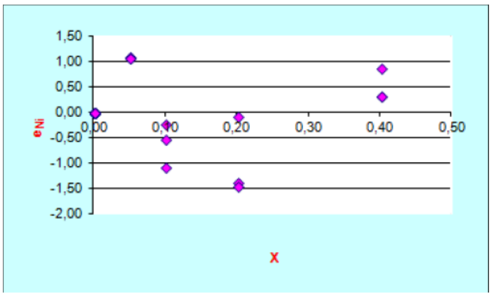

Normalized residuals

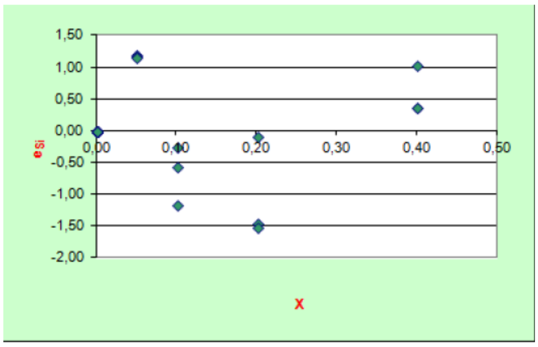

Standardized residuals

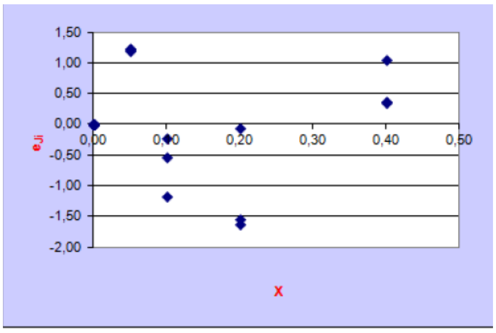

Studentized residuals

Residuals (+)	6
Residuals (-)	6
Sequences number	7
Reference parameter	7
Correction factor	3.272727

h_i (leverages coefficient); e_i (absolute residuals); e_{Ni} (normalized residuals); e_{Si} (studentized residuals); e_{ji} (standardized residuals)

Figure 3. Shapiro-Wilk test (5%significance levels).

**Red wine vinegar**	
Community method<br>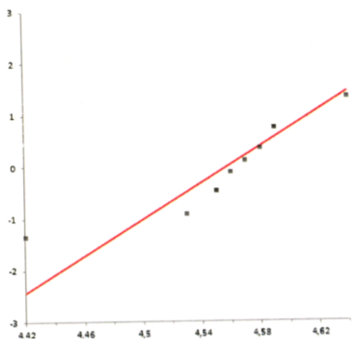	Enzymatic method<br>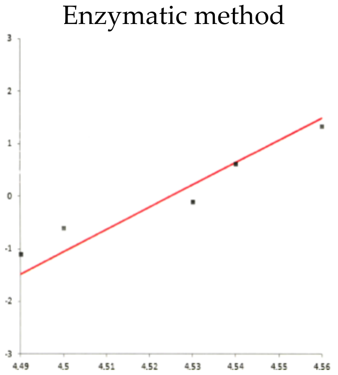
W statistic: 0.86<br>*p*-value: 0.0701<br>Do not reject the null hypothesis	W statistic: 0.87<br>*p*-value: 1.1093<br>Do not reject the null hypothesis
**Withe wine vinegar**	
Community method<br>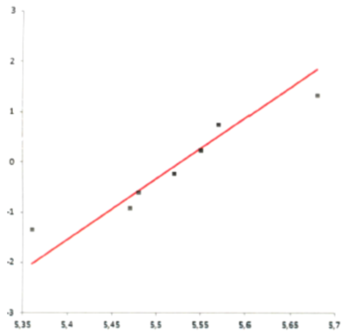	Enzymatic method<br>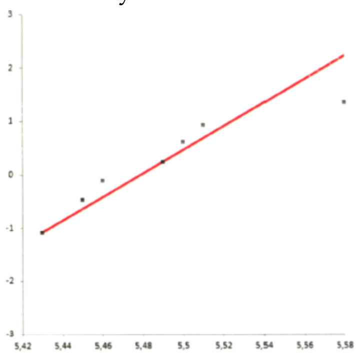
W statistic: 0.94<br>*p*-value: 0.5540<br>Do not reject the null hypothesis	W statistic: 0.89<br>*p*-value: 1.843<br>Do not reject the null hypothesis
**Balsamic vinegar**	
Community method<br>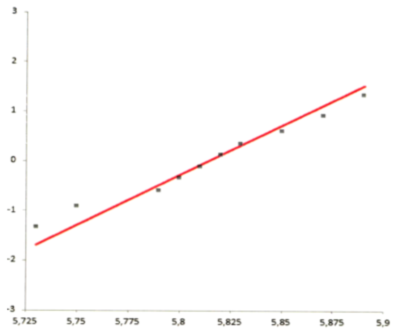	Enzymatic method<br>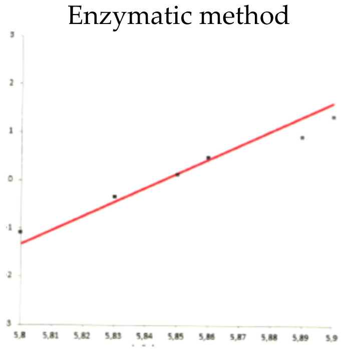
W statistic: 0.98<br>p value: 0.9594<br>Do not reject the null hypothesis	W statistic: 0.93<br>p value: 0.4984<br>Do not reject the null hypothesis

y=

normal theoretical quartile

x= concentration

Figure 4 Balsamic wine vinegar accuracy statistical analyses.

	**Community method**	**Enzymatic method**
1	5.73	5.8
2	5.81	5.86
3	5.89	5.86
4	5.79	5.89
5	5.8	5.8
6	5.82	5.9
7	5.87	5.83
8	5.85	5.85
9	5.75	5.83
10	5.83	5.83

t Student

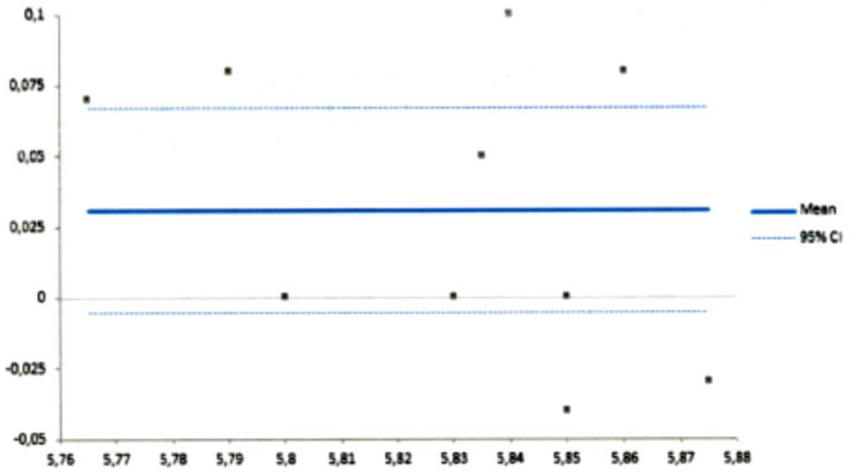

y= concentration official meth/enzymatic meth

x= $\frac{\text{concentration official meth/enzymatic meth}}{2}$

Mean difference: -0.031
95%Cl: -0.005 to 0.067
SE: 0.0160
Student test
Hypothesized difference: 0
t statistic: 1.94
DF: 9
p-value: 0.0849
Do not reject the null hypothesis at the 5%significance level

Figure 5. Red wine vinegar accuracy statistical analyses.

	Community method	Enzymatic method
1	4.55	4.53
2	4.42	4.54
3	4.56	4.54
4	4.59	4.54
5	4.53	4.53
6	4.64	4.56
7	4.59	4.5
8	4.58	4.49
9	4.55	4.53
10	4.57	4.49

t Student

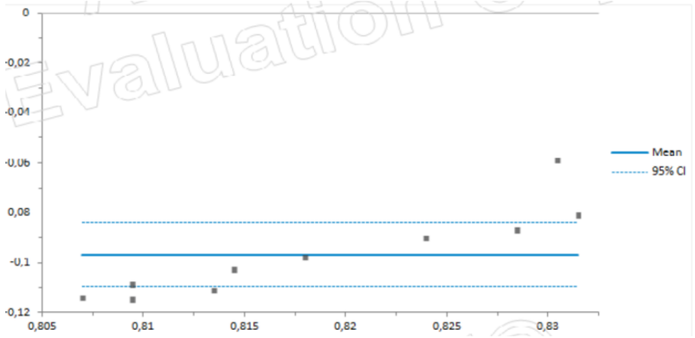

y= concentration official meth/enzymatic meth

$$x = \frac{\text{concentration official meth/enzymatic meth}}{2}$$

Mean difference: 0.033
95%Cl: -0.012 to 0.078
SE: 0.0201
Student test
Hypothesized difference: 0
t statistic: 1.65
DF: 9
p-value: 0.1343
Do not reject the null hypothesis at the 5%significance level

Figure 6. White wine vinegar accuracy statistical analyses.

	Community method	Enzymatic method
1	5.48	5.43
2	5.52	5.43
3	5.55	5.45
4	5.57	5.46
5	5.36	5.45
6	5.55	5.51
7	5.47	5.5
8	5.552	5.58
9	5.57	5.49
10	5.68	5.49

t Student

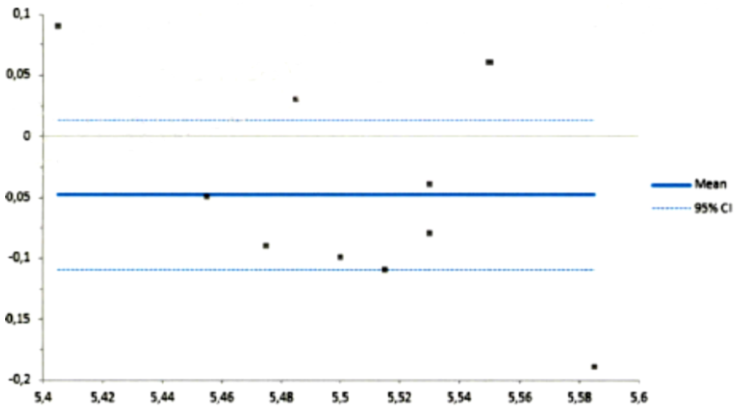

y= concentration official meth/enzymatic meth

x= $\frac{\text{concentration official meth/enzymatic meth}}{2}$

Mean difference: -0.048
95%Cl: -0.109 to 0.013
SE: 0.0272
Student test
Hypotesized difference: 0
t statistic: -1.77
DF: 9
p-value: 0.1109
Do not reject the null hypothesis at the 5%significance level
